# Supplementary material for: Molecular basis for N-terminal acetylation by human NatE and its modulation by HYPK
Source: Nat Commun. 2020 Feb 10;11:818. doi: 10.1038/s41467-020-14584-7 (PMC7010799; doi:10.1038/s41467-020-14584-7)
Supplement: Supplementary file 1 — Supplementary Information [file 41467_2020_14584_MOESM1_ESM.pdf]

Supplementary Information

Molecular basis for N-terminal acetylation by human NatE and its modulation by HYPK

Deng. et al

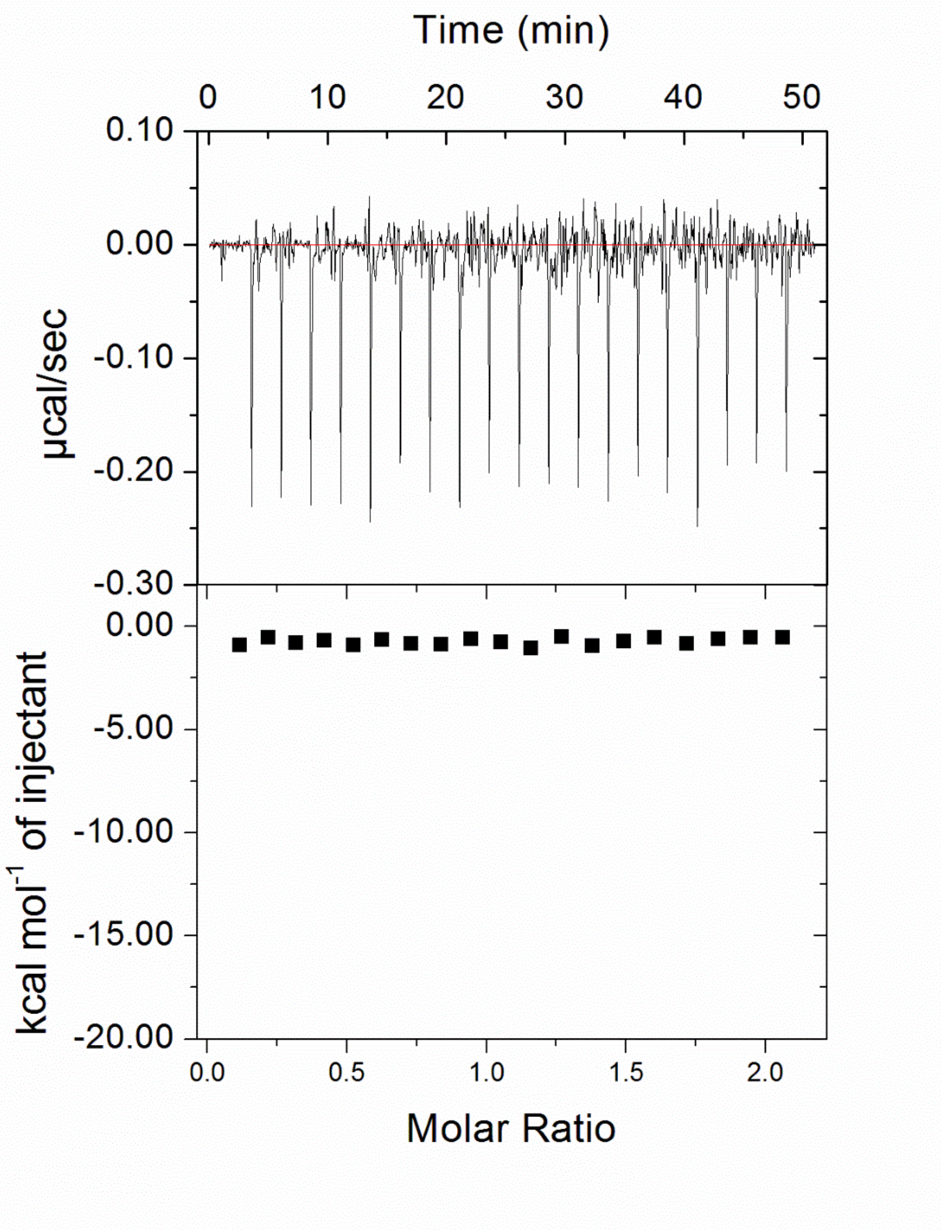

Supplementary Figure 1., Related to Fig.1 c-e, ITC data of MBP titrated into hNatA.

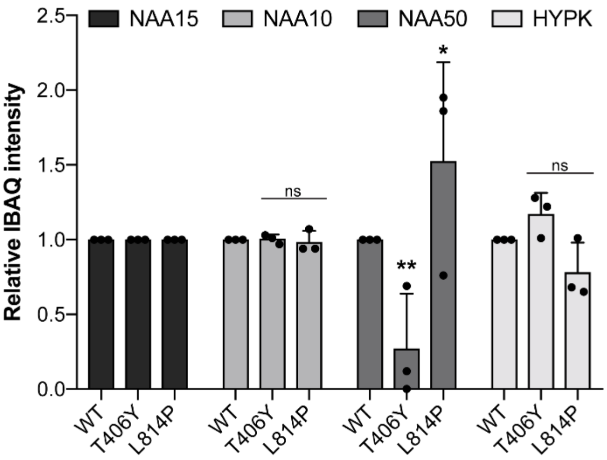

Supplementary Figure 2., Related to Fig. 3a. Mass spectrometry analysis of NAA15 and its variants. Bar graph displaying the mean relative IBAQ intensity of NatA components from three independent mass spectrometry analyses of immunoprecipitated NAA15 variants. The IBAQ intensities of each component were normalized to the IBAQ intensity of NAA15 in the respective sample and to the corresponding NatA WT protein. Source data are provided as a Source Data file Error bars are reported in SD with n = 3 biologically independent experiments. Significance was determined using 2way ANOVA test (**p = 0.0014, *p = 0.00198).

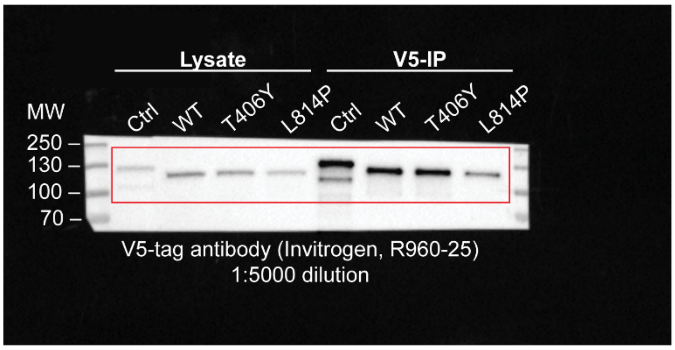

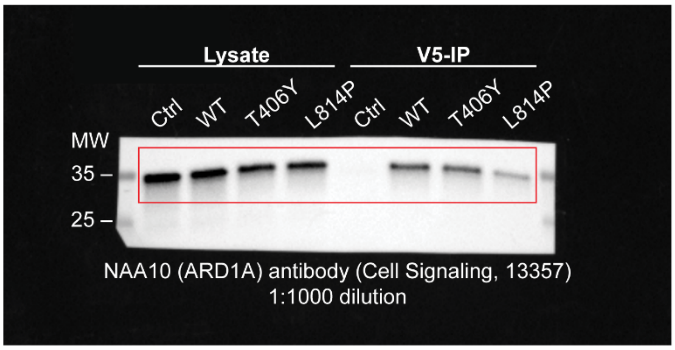

Supplementary Figure 3., Related to Fig. 3b. Uncropped Western Blot.

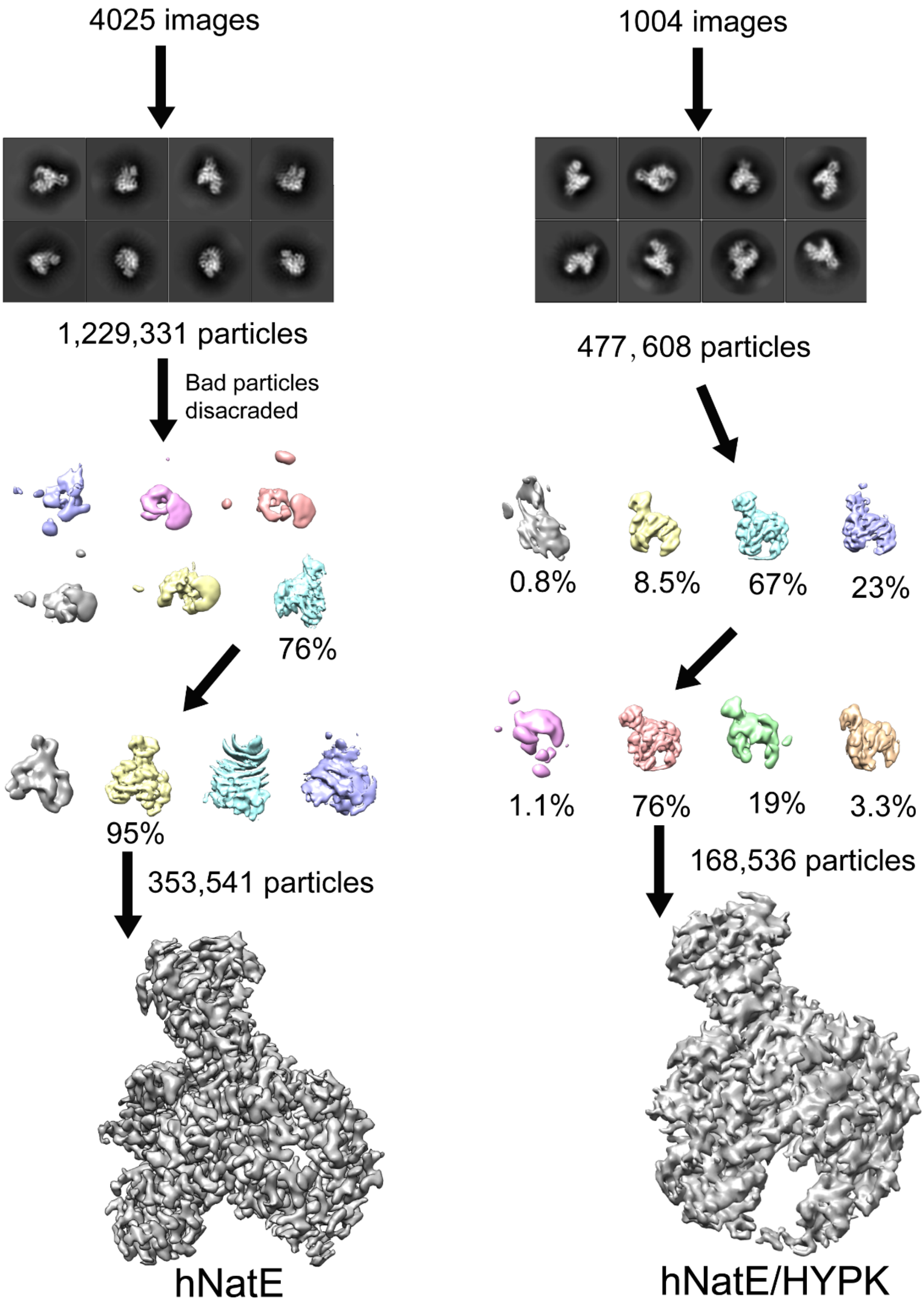

Supplementary Figure 4. 2D and 3D classification scheme for EM maps determination. Particles were picked automatically, and 2D classification was used to discard bad particles. Only good particles after 3D classification were used for refinement.

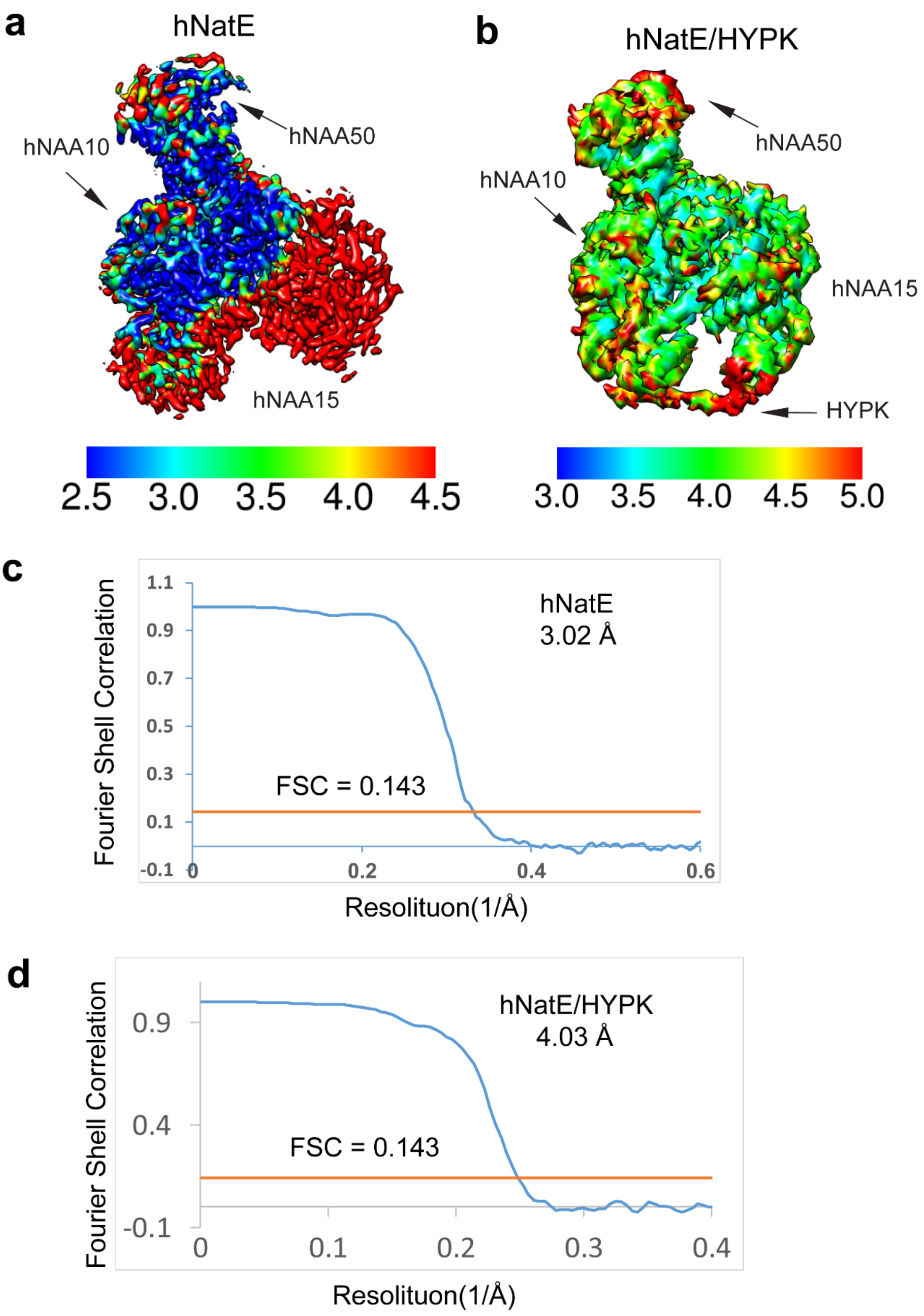

Supplementary Figure 5. Local resolution and FSC curve for EM maps. Local resolution maps of hNatE and hNatE/HYPK complexes are shown in **(a)** and **(b)**, respectively. FSC curves of hNatE and hNatE/HYPK complexes are shown in **(c)** and **(d)**, respectively.

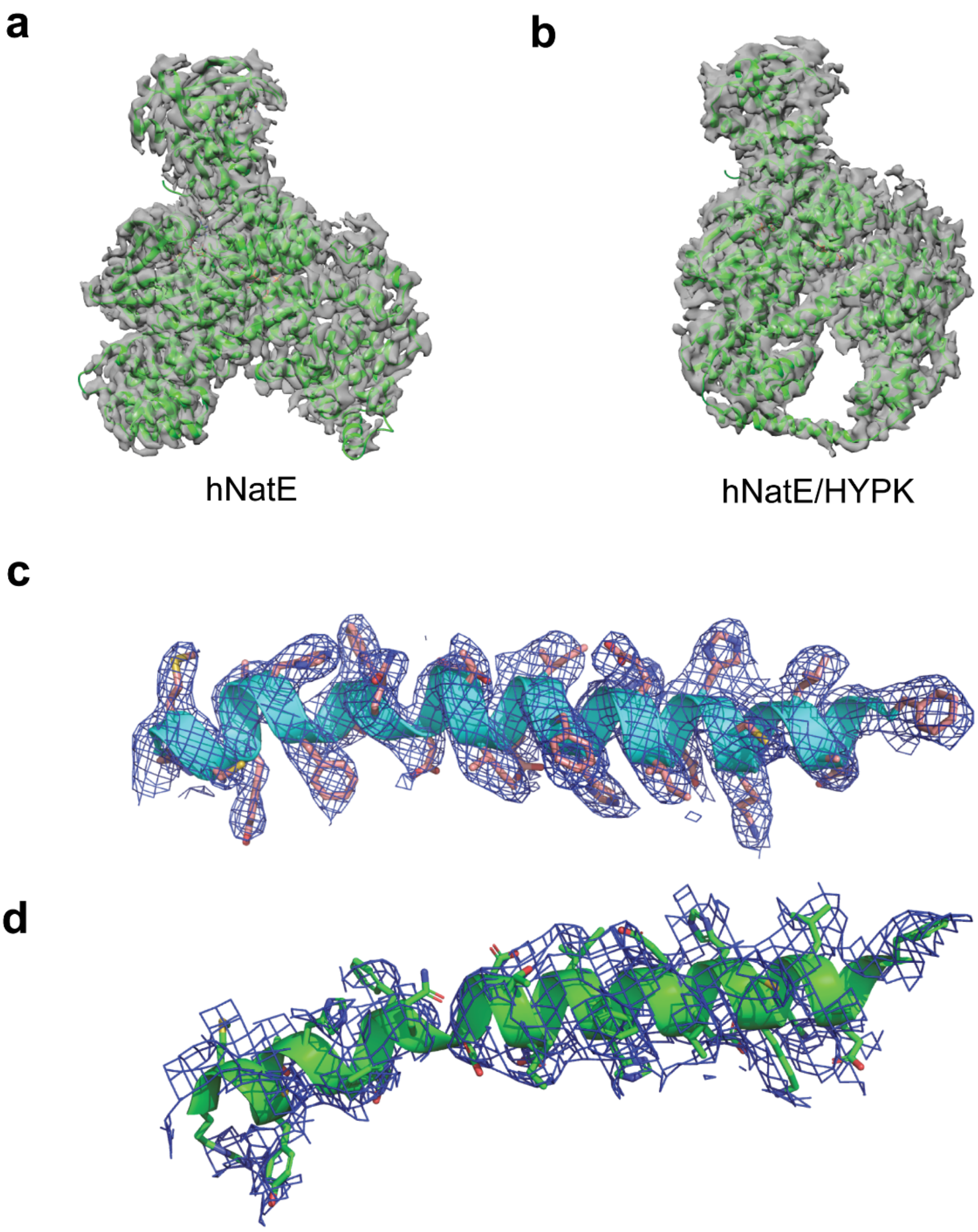

Supplementary Figure 6. Fit of atomic model into the Cryo-EM map. Atomic model of hNatE **(a)** and hNatE/HYPK **(b)** were fitted into the corresponding Cryo-EM map with contour level of 0.012 and 0.015, respectively. **(c)** A helical segment from hNatE model was fitted into the EM density. (d) A helical segment from hNatE/HYPK model was fitted into the EM density. Contour levels for (c) and (d) are 4 sigma.

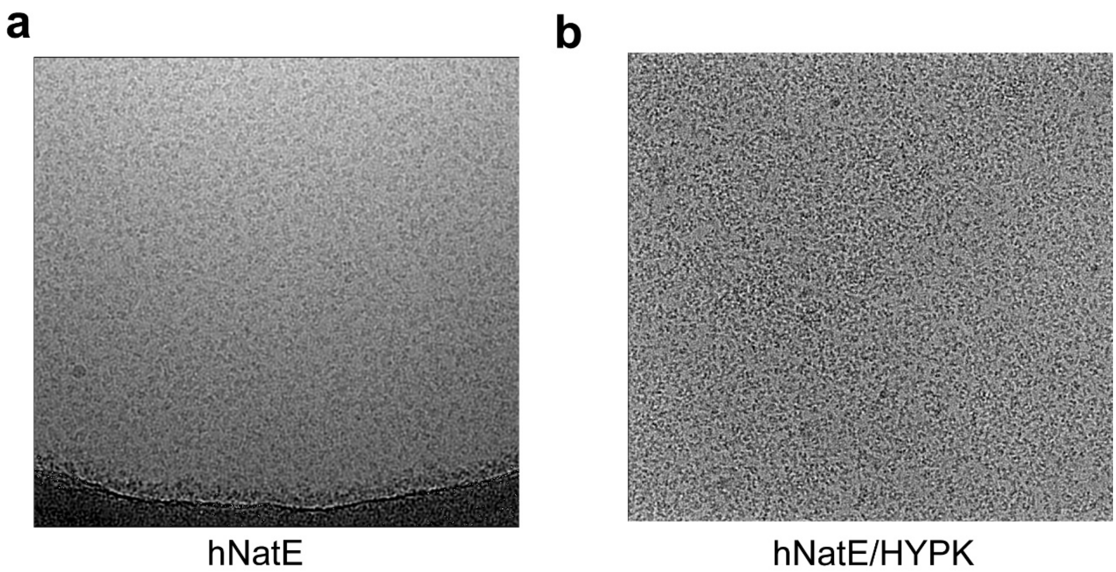

Supplementary Figure 7. Representative micrographs with particles. Representative micrograph of hNatE **(a)** and hNatE/HYPK **(b)** for corresponding Cryo- EM map determination.
