# Supplementary material for: Molecular basis for N-terminal acetylation by human NatE and its modulation by HYPK
Source: Nat Commun. 2020 Feb 10;11:818. doi: 10.1038/s41467-020-14584-7 (PMC7010799; doi:10.1038/s41467-020-14584-7)
Supplement: Supplementary file 3 — Description of Additional Supplementary Files [file 41467_2020_14584_MOESM3_ESM.pdf]

Description of Additional Supplementary Files

Supplementary Data 1. Mass spectrometry analysis of the V5-immunoprecipitates of the hNAA15-wt-V5, hNAA15-T406Y-V5 and hNAA15-L814P-V5 mutants. For relative quantification of the hNatA proteins, the IBAQ intensities of the hNatA components in each sample were normalized to the IBAQ intensity of hNAA15 in the respective sample and to the corresponding protein in the WT sample
