# Supplementary material for: Molecular basis for N-terminal acetylation by human NatE and its modulation by HYPK
Source: Nat Commun. 2020 Feb 10;11:818. doi: 10.1038/s41467-020-14584-7 (PMC7010799; doi:10.1038/s41467-020-14584-7)
Supplement: Supplementary file 5 — Reporting Summary [file 41467_2020_14584_MOESM5_ESM.pdf]

nature research

Corresponding author(s): Ronen Marmorstein

Last updated by author(s): Dec 19, 2019

Reporting Summary

Nature Research wishes to improve the reproducibility of the work that we publish. This form provides structure for consistency and transparency in reporting. For further information on Nature Research policies, see Authors & Referees and the Editorial Policy Checklist .

Statistics

For all statistical analyses, confirm that the following items are present in the figure legend, table legend, main text, or Methods section.

n/a	Confirmed	
☐	☒	The exact sample size (*n*) for each experimental group/condition, given as a discrete number and unit of measurement
☐	☒	A statement on whether measurements were taken from distinct samples or whether the same sample was measured repeatedly
☒	☐	The statistical test(s) used AND whether they are one- or two-sided<br>*Only common tests should be described solely by name; describe more complex techniques in the Methods section.*
☒	☐	A description of all covariates tested
☒	☐	A description of any assumptions or corrections, such as tests of normality and adjustment for multiple comparisons
☐	☒	A full description of the statistical parameters including central tendency (e.g. means) or other basic estimates (e.g. regression coefficient) AND variation (e.g. standard deviation) or associated estimates of uncertainty (e.g. confidence intervals)
☒	☐	For null hypothesis testing, the test statistic (e.g. *F*, *t*, *r*) with confidence intervals, effect sizes, degrees of freedom and *P* value noted<br>*Give P values as exact values whenever suitable.*
☒	☐	For Bayesian analysis, information on the choice of priors and Markov chain Monte Carlo settings
☒	☐	For hierarchical and complex designs, identification of the appropriate level for tests and full reporting of outcomes
☒	☐	Estimates of effect sizes (e.g. Cohen's *d*, Pearson's *r*), indicating how they were calculated

Our web collection on statistics for biologists contains articles on many of the points above.

Software and code

Policy information about availability of computer code

Data collection	EM-Tools
Data analysis	MotionCor2, Gctf v1.06, RELION 3.0, COOT 0.8.9.1, Chimera 1.12, Phenix, Pymol , GraphPad Prism 5, Perseus software v. 1.6.5.0 (Mass spectrometric analysis)

For manuscripts utilizing custom algorithms or software that are central to the research but not yet described in published literature, software must be made available to editors/reviewers. We strongly encourage code deposition in a community repository (e.g. GitHub). See the Nature Research guidelines for submitting code & software for further information.

Data

Policy information about availability of data

All manuscripts must include a data availability statement. This statement should provide the following information, where applicable:

- Accession codes, unique identifiers, or web links for publicly available datasets
- A list of figures that have associated raw data
- A description of any restrictions on data availability

The cryo-electron microscopy map for the hNatE and hNatE/HYPK and the respective atomic coordinates have been deposited in the EMDataBnak and ProteinDataBank, with accession codes of EMD-20442 and 6PPL for hNatE, respectively, and with EMD-20501 and 6PW9 for hNatE/HYPK.

Field-specific reporting

Please select the one below that is the best fit for your research. If you are not sure, read the appropriate sections before making your selection.

Life sciences study design

All studies must disclose on these points even when the disclosure is negative.

Sample size	Fluorescence Polarization binding assay, ITC experiments, Mass Spectrometry, and Acetyltransferase Activity Assays were performed three times.
Data exclusions	no data were excluded from analysis
Replication	All biochemical and biophysical experiments were successfully reproduced. Cryo-EM data collection and analysis was performed once.
Randomization	no randomization required
Blinding	blinding was not required

Reporting for specific materials, systems and methods

We require information from authors about some types of materials, experimental systems and methods used in many studies. Here, indicate whether each material, system or method listed is relevant to your study. If you are not sure if a list item applies to your research, read the appropriate section before selecting a response.

Materials & experimental systems

n/a	Involved in the study	
☐	☒	Antibodies
☐	☒	Eukaryotic cell lines
☒	☐	Palaeontology
☒	☐	Animals and other organisms
☒	☐	Human research participants
☒	☐	Clinical data

Methods

n/a	Involved in the study	
☒	☐	ChIP-seq
☒	☐	Flow cytometry
☒	☐	MRI-based neuroimaging

Antibodies

Antibodies used	V5-tag mouse monoclonal antibody
Validation	purchased from Invitrogen (R960-25)

Eukaryotic cell lines

Policy information about cell lines

Cell line source(s)	SF9, HeLa cells
Authentication	None of the cell lines used were authenticated.
Mycoplasma contamination	The cell lines were not test for mycoplasma contamination
Commonly misidentified lines (See ICLAC register)	*Name any commonly misidentified cell lines used in the study and provide a rationale for their use.*
